# Qualitative vs. Quantitative Damage: Identifying Critical Susceptibility Interval of Common Bean to *Euschistus heros* (Hemiptera: Pentatomidae)

**DOI:** 10.3390/insects17040404

**Published:** 2026-04-09

**Authors:** Bruna Teixeira Baixo, Adriano Thibes Hoshino, Luciano Mendes de Oliveira, Millena dos Santos Rodrigues, Helter Carlos Pereira, Ayres de Oliveira Menezes Junior, Humberto Godoy Androcioli

**Affiliations:** 1 Department of Agronomy, State University of Londrina, Londrina 86057-970, PR, Brazil; brunatbaixo@gmail.com (B.T.B.); ayres@uel.br (A.d.O.M.J.); 2 Department of Agronomy, Federal University of Paraná, Jandaia do Sul Campus, Jandaia do Sul 86900-000, PR, Brazil; adriano.hoshino@ufpr.br; 3 Laboratory of Entomology, Rural Development Institute of Paraná—IAPAR-EMATER, Londrina 86047-902, PR, Brazil; lucianomendes.oliveira@idr.pr.gov.br (L.M.d.O.); millena.rodrigues@hotmail.com (M.d.S.R.); helterpereira@idr.pr.gov.br (H.C.P.)

**Keywords:** *Phaseolus vulgaris*, neotropical brown stink bug, reproductive structure abortion, grain type, carioca type bean, phytophagous insect, growth habit

## Abstract

This study evaluated the susceptibility of common bean (*Phaseolus vulgaris* L.) cultivars to *Euschistus heros* feeding across various phenological stages. Three cultivars (IPR Curió, IPR Sabiá, and IPR Urutau) were infested with 0.5 insects per plant for eight days starting at anthesis and 8, 16, 24, 32, and 40 days after flowering (DAF) using a randomized block design with five replicates. *E. heros* did not significantly impact grain yield or reproductive abscission, except for the IPR Curió cultivar during flowering, which demonstrated substantial qualitative damage. Feeding injury resulted in increased grain punctures and the grading of commercial classification to Type 2. The most critical susceptibility period occurred during the grain-filling stages (16–24 DAF). IPR Curió was the most sensitive cultivar, exhibiting Type 2 status at both 16 and 24 DAF. These findings demonstrate that although common beans exhibit quantitative tolerance to *E. heros* at the tested density, qualitative damage during grain development significantly compromises marketability and value. Integrated Pest Management (IPM) should prioritize protecting the crop during mid-to-late reproductive stages to ensure that grain quality standards are met.

## Simple Summary

This study researched how the neotropical brown stink bug affects common beans during different stages of growth. Three different bean varieties were tested in a greenhouse to see when they were most susceptible to damage. The stink bugs did not reduce the total amount (weight) of beans harvested, nor did they cause the plants to abort their flowers. However, they did cause significant quality problems. The bugs pierce into the developing beans, leaving punctures. These marks make the beans look unattractive, which lowers their grade and market value. The most critical time for damage is during the grain-filling stage, specifically between 16 and 24 days after the flowers first bloom. During this stage, the beans were graded from the best quality (Type 1) to a lower grade (Type 2). Among the varieties tested, the IPR Curió was the most sensitive to these attacks.

## 1. Introduction

The common bean (*Phaseolus vulgaris* L.) is the primary legume consumed globally, serving as a vital source of proteins, vitamins, and minerals. Beyond its nutritional profile, it plays a fundamental role in food security, particularly within developing nations [1,2]. In 2024, global production reached approximately 30.3 million tons across 32.7 million hectares [3]. Despite its importance, the crop is susceptible to various phytosanitary challenges that compromise both yield and grain quality [4,5,6]. Among these threats, insect pests that directly damage the grains are of particular concern, as visual appearance is a critical attribute for marketability [7,8,9].

Phytophagous stink bugs (Hemiptera: Pentatomidae) are among the most significant pests during the reproductive stages of several legumes [10,11,12]. These insects insert their piercing–sucking mouthparts into pods and seeds, injecting digestive enzymes that cause tissue degradation and physiological alterations [13,14]. Attacked grains often become shriveled, underdeveloped, and discolored [15]. Furthermore, infestation can lead to pod abortion, reduced oil content, and physiological disorders such as delayed maturation, like green stem syndrome (GSS), as well as hindered seed germination and vigor [16,17]. The magnitude of this damage is intrinsically linked to the plant’s phenological stage at the time of infestation, with reproductive stages being the most vulnerable [10,18].

Among the pentatomid species, *Euschistus heros* (Fabricius, 1798), or the neotropical brown stink bug as it is known in Brazil, is a key pest in soybean [*Glycine max* (L.) Merrill] cultivation [19,20,21,22]. However, population outbreaks are increasingly reported in common bean crops, primarily driven by the migration of individuals from senescing soybean fields or their emergence from overwintering sites in the spring [23,24]. This scenario is exacerbated in regions where beans are cultivated in up to three distinct seasons per year, allowing for a continuous succession of host plants and the uninterrupted biological cycle of the insect [25,26].

In soybeans, the critical period for stink bug occurrence spans from late pod development to early grain filling, during which the highest economic damage occurs [27,28]. During this interval, rigorous monitoring is essential to prevent populations from exceeding the Economic Injury Level (EIL), thereby safeguarding yield and seed quality [15,29,30]. Parameters such as the Control Threshold (CT) and critical infestation periods are well-established and frequently updated for soybean [20,31,32], but significant knowledge gaps remain for the common bean, especially regarding modern cultivars with diverse growth habits and phenological traits. Consequently, this study tests the hypothesis that the critical susceptibility window varies according to the common bean phenological stage.

Therefore, this study aimed to assess how varying phenological stages influence the susceptibility of three common bean cultivars to *E. heros* feeding, thereby providing essential data to refine Integrated Pest Management (IPM) strategies for this crop.

## 2. Materials and Methods

### 2.1. Study Site and Environmental Conditions

The study was conducted from April to July 2021 in a greenhouse at the Experimental Station of the Institute of Rural Development of Paraná (IDR-Paraná) in Londrina, PR, Brazil (23°21′33″ S, 51°09′49″ W). To prevent plant etiolation, a 14 h photophase was maintained using three 400 W HQI-T metal halide lamps, with a luminous of 35,000 lumens and a color temperature of 5000 K (Osram, São Paulo, Brazil) Environmental variables were monitored using data loggers (AZ Instrument Corp model 8829, Taichung, Taiwan), which recorded a mean temperature of 24.6 °C (range: 17.9–35.0 °C) and a mean relative humidity (RH) of 62% throughout the experimental period.

### 2.2. Insect Rearing

Specimens of *E. heros* were obtained from a laboratory colony maintained at IDR-Paraná under controlled conditions (28 ± 4 °C; 70 ± 20% RH). The insects were reared on a diet of common beans pods (*Phaseolus vulgaris*), soybean grains (*Glycine max*), peanuts (*Arachis hypogaea* L.), and maize sprouts (*Zea mays* L.). Only 7-day-matured adult fecundated females were selected, from a mixed-sex population, for the experiment, to ensure uniform age and feeding vigor.

### 2.3. Experimental Design and Infestation Procedure

The experiment studied three common bean cultivars: IPR Curió (carioca type, determinate and early maturing cycle), IPR Sabiá (carioca type, indeterminate and medium maturing cycle), and IPR Urutau (black type, indeterminate and medium maturing cycle). Sowing was performed in 5 L pots containing two plants each, spaced 10 cm apart and managed according to recommended agronomic practices for the crop. During the vegetative stage (starting at V3), yellow sticky traps (10 cm × 25 cm) were placed between pots to monitor and capture any transient greenhouse pests.

Prior to anthesis, the pots were fitted with cylindrical wire frames (0.5 m diameter, 1.5 m height) covered with fine tulle mesh (5 × 5 mm mesh size). Each cage featured a longitudinal zipper to facilitate insect introduction and daily mortality checks. The base of the mesh was secured with elastic bands to prevent insect escape or the entry of external pests.

Plants were infested with a density of 0.5 insects per plant at six distinct phenological stages: anthesis (R6) and 8, 16, 24, 32, and 40 days after flowering (DAF). The specific phenological status for each cultivar at the time of infestation is detailed in Table 1.

For each treatment, the insects remained confined for eight days. Cages were inspected every 48 h, eggs were discarded and dead insects were replaced to maintain constant infestation pressure. A non-infested control group was also maintained under identical caged conditions. Following each 8-day period, insects were manually removed, and the plants remained caged until harvest to ensure all treatments developed under uniform environmental conditions.

### 2.4. Harvest and Grain Classification

Harvesting occurred when 90% of the pods reached physiological maturity (dry pods). Grains were initially categorized as healthy, aborted, or shriveled. Subsequently, each class was sorted using oblong-hole sieves (sizes: 5.0, 4.5, 4.0, 3.75, and 3.5 × 22 mm). Following the protocol of the IDR-Paraná Seed laboratory, grains retained by the 3.75 × 22 mm sieve and larger were classified as commercial-grade. Grains that fall below the 3.75 mm sieves are usually discarded during mechanical harvest and thus have no commercial value.

Samples were stored in labeled paper bags and dried in a forced air oven (SOLIDSTEEL model: 1600-110) (São Paulo, Brazil) at 45 °C until reaching a uniform moisture content of 14%, verified using a grain moisture meter (Gehaka model G939IP, São Paulo, Brazil). After drying, the dry mass was recorded, and commercial grains were further subdivided into healthy grains and those exhibiting feeding punctures.

Finally, commercial grain quality was graded based on the percentage of minor defects (immature, bruised, or damaged grains) in accordance with the Instrução Normativa #12/2008 of the Brazilian Ministério da Agricultura e Pecuária (MAPA) (Table 2).

### 2.5. Variables Evaluated

Data collection was divided into the reproductive and post-harvest stages of the crop. During the reproductive period, the number of aborted reproductive structures (flowers and pods) was monitored and recorded every 48 h. This same protocol was applied to the non-infested control group to determine whether the timing of *E. heros* infestation significantly influenced the rate of reproductive abortion.

Post-harvest evaluations included the following variables: aborted and shriveled grains (%); commercial grain yield (g.plant^−1^); commercial grains with feeding punctures (%); and commercial grain classification (Type) based on physical quality standards.

### 2.6. Statistical Analysis

The experiment followed a randomized block design with five replicates. Student’s *t*-test was employed to compare the number of aborted structures between each treatment and the control group. All other quantitative datasets were subjected to Analysis of Variance (ANOVA). When significant differences were detected, treatment means were compared using Tukey’s Honestly Significant Difference test (α ≤ 0,05). Percentage data were transformed using square root transformation, where k = 0.5. All statistical analyses were performed using SASM-Agri (version 1.0) and BioEstat (version 5.0) software [34,35].

## 3. Results

**Figure 1:**
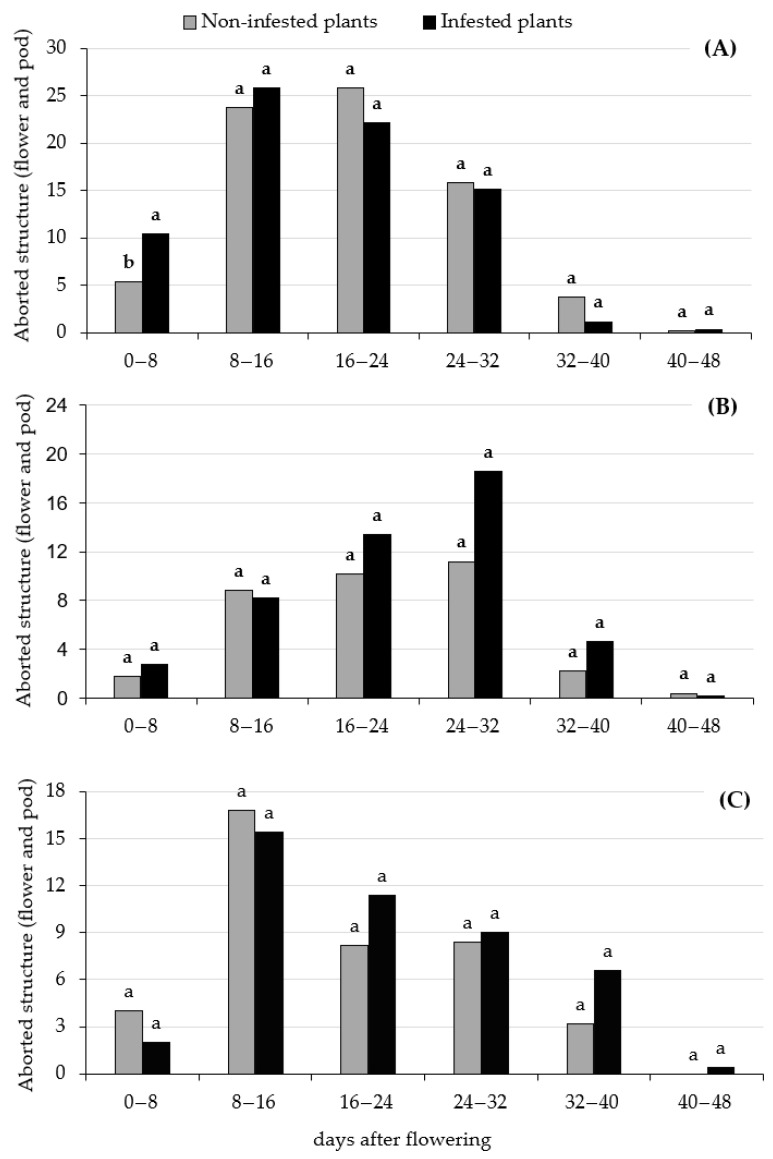
Average aborted reproductive structures (flower and pod) of the common bean cultivars IPR Curió (**A**), IPR Urutau (**B**) and IPR Sabiá (**C**). Comparison between plants without infestation (grey columns) and plants with *Euschistus heros* infestations (black columns) on different days after flowering. Columns with the same letter do not significantly vary following *t*-test (α = 0.05).

With the exception of the anthesis (IF) stage for the cultivar IPR Curió, infestation by *E. heros* at a density of 0.5 insects per plant did not affect flower abortion and pods. No statistical differences were observed between the non-infested control and the infestation treatments (*p* > 0.05) (Figure 1).

Regarding the percentage of aborted grains, a difference was observed only for IPR Sabiá when infested at IF and 40 DAF, with values significantly higher than at 16 and 24 DAF (*p* = 0.01) (Table 3). Notably, the percentage of shriveled grains did not differ across any of the treatments or cultivars, suggesting that at this density, the insect’s impact is more localized to specific stages rather than a general reduction in grain development.

The presence of *E. heros* at 0.5 insects per plant across different phenological stages did not result in a reduction in the weight of commercial grains produced (Table 3). However, grain quality was markedly affected. The punctured commercial grain % (PCG) showed differences for IPR Curió (*p* = 0.05) and IPR Urutau (*p* = 0.04). For IPR Curió, the highest incidence of punctured seeds occurred during infestations at 16 and 24 DAF, coinciding with the grain-filling stage. For IPR Urutau, the highest percentage was recorded at 16 DAF, which spans the period from late pod development to the onset of grain filling, significantly higher than percentages found at 40 DAF, which were null.

The quality of the harvested beans was categorized into Type 1 and Type 2 based on physical defects. IPR Curió was graded as Type 2 when infested at 16 and 24 DAF. IPR Sabiá reached Type 2 classification at 24 DAF. IPR Urutau was classified as Type 2 at 16 DAF.

In all three cultivars, the reduction in commercial grade (Type 2 classification) occurred exclusively during the grain-filling stages, highlighting this as the most critical window for qualitative damage.

## 4. Discussion

High rates of reproductive structure abortion are common in common beans, often ranging from 60% to 80% of total flowers produced [36]. In this study, the cultivar IPR Curió exhibited high abscission rates, likely due to its determinate growth habit. In contrast to indeterminate cultivars, which continue vegetative growth and flower production simultaneously, determinate cultivars cease vegetative growth at flowering; this, however, does not influence the potential productivity of the IPR Curió cultivar [24]. This shifts the plant’s physiological focus entirely towards flowering, an excess of which triggers greater flower and pod abortion as a mechanism to balance the source–drain relationship [37]. Furthermore, intense flowering accelerates respiration rates, creating a high demand for carbohydrates that may further reduce the viability of young pods and flowers [36]. In the conditions of the present studies, the IPR Sabiá and IPR Urutau cultivars’ abortion rate was not affected by *E. heros* feeding, independent of the infestation moment. Further research is necessary to establish phytophagous Pentatomidae damage potential towards indeterminate common bean cultivars.

Infestation by *E. heros* did not reduce the total weight of commercial grains. This occurred despite using a population density higher than the recommended action thresholds for both common bean and soybean [20,24,38,39]. These findings align with previous field studies on IPR Curió, which also reported no quantitative yield losses under *E. heros* pressure [24]. Similar trends have been observed in soybeans infested with the brown marmorated stink bug (*Halyomorpha halys* Stål) (Hemiptera: Pentatomidae), where yield loss was not associated with the differing insect densities [40].

The absence of quantitative damage may be attributed to plant compensatory mechanisms. Legumes can often increase the size of unaffected seeds to compensate for those lost or damaged during early development [41,42]. Moreover, stink bug damage severity depends on the phenological stage, population density, and infestation duration [40,43]. For instance, studies on soybean at the R6 stage (fully grained) showed that while yield remained stable even after 21 days of infestation, seed quality (viability and vigor) significantly withered [17].

It is important to highlight that different Pentatomidae specimens present varying damage potential towards pods and seeds [44]. *Piezodorus guildinii* (Westwood) has the highest damage potential towards soybean, given its greater salivary enzyme toxicity and tissue penetration capability, when compared to *Diceraeus melacanthus* (*Dallas*), *Nezara viridula* (L.) and *E. heros* [13]. The migration of these stink bugs from soybean fields towards common bean fields is well documented [23,24]. However, these specimens’ effects on common bean grains are relatively unknown; thus, further research is recommended.

The severity of pentatomid damage is intrinsically linked to the grain development stage [10]. Feeding during early pod and seed development typically leads to seed abortion, while feeding during the grain-filling stage results in shriveled, deformed, or maldeveloped grains. Once grains reach physiological maturity, they become less suitable for feeding, leading only to minor deformations or superficial punctures [10,18,30].

The current study supports this, as the incidence of punctured grains decreased once the plants reached physiological maturity (40 DAF). This suggests that the Control Threshold (CT) and Economic Injury Level (EIL) could potentially be elevated during the final stages of crop maturation [17,45,46].

While yield quantity was preserved, qualitative damage was significant. All evaluated cultivars were graded to Type 2 status when infested during the grain-filling stages, with IPR Curió demonstrating the highest susceptibility during certain infestation moments (16 and 24 DAF). Similar qualitative declines have been documented in soybeans infested during early reproductive stages [43].

Beyond the immediate visual defects, *E. heros* injury can compromise long-term storage quality [47]. Attacked grains exhibit higher fermentation rates and increased acidity levels, which negatively impact shelf life and commercial value [48]. Bean visual aspect constitutes the fundamental choice criterion for consumers [49]. Commercial quality is directly tied to visual aspects: qualities like color, sheen, shape and size [50,51]. Thus, the increase in damaged grains originating from *E. heros* feeding may considerably reduce commercial value.

The migration of *E. heros* from soybean-harvested to common bean-cultivated fields may cause considerable commercial grain depreciation [23,24]. The damage potential is greater in regions where three common bean harvests are possible [25,26]. Future studies evaluating this pest’s ecology and behavior are essential for more environmentally safe and ecological IPM programs and control decision making.

Infestation by *E. heros* at a density of 0.5 insects per plant did not induce the abortion of reproductive structures across the evaluated common bean cultivars, with the exception of IPR Curió during anthesis. Furthermore, the timing of infestation across various phenological stages had no effect on the percentage of aborted or shriveled grains, nor did it impact the final commercial yield, indicating that this pest density does not result in quantitative losses.

In contrast, qualitative losses were observed, characterized by an increased incidence of feeding punctures and the subsequent Type 2 commercial grading of grains. These qualitative impacts were most pronounced during the grain-filling phase, specifically at 16 and 24 DAF for IPR Curió, 24 DAF for IPR Sabiá, and 16 DAF for IPR Urutau. Among the evaluated genotypes, IPR Curió demonstrated the highest susceptibility to *E. heros* injury, reaching Type 2 status across two distinct infestation windows and exhibiting a significantly higher percentage of punctured commercial grains.

## Figures and Tables

**Table 1:** Common bean phenological condition for the IPR Curió, IPR Sabiá and IPR Urutau cultivars infested with *Euschistus heros* adults varying days after flowering (DAF).

Infesting Moments	Common Bean Phenological Condition
Initial flowering (IF)	Anthesis
8 DAF	Pods in final maturing period (>3 cm) (PFM)
16 DAF	Initial grain filling (IGF)
25 DAF	Final grain filling (FGF)
32 DAF	Physiological maturity period (PMP) *
40 DAF	Physiological maturity period (PMP) *

* At least 50% of plants have reached maturity.

**Table 2:** Defect tolerance expressed in % per weight and respective type sorting for common bean (*Phaseolus vulgaris*).

Type Sorting	Total Minor Defects
Type 1	Zero to 2.50%
Type 2	>2.50% to 6.50%
Type 3	>6.50% to 16.00%
Atypical	>16.00%

Source: Instrução Normativa #12/2008 do Ministério da Agricultura e Pecuária [33].

**Table 3:** Aborted (AG) and shriveled (SG) grain percentage, commercial grain weight (CGW), punctured commercial grain percentage (PCG) and grain type classification, considering minor damages, evaluated for the common bean cultivars IPR Curió, IPR Sabiá and IPR Urutau in varying moments of *Euschistus heros* (0.5 stink bug/plant) infestations during the common bean reproductive stage in controlled conditions.

Cultivar	Infestation Moment	AG (%)	SG (%)	CGW (g.Plant^−1^)	PCG (%)	Type
IPR Curió	Control	23.18 ± 3.78 ^NS^	4.12 ± 2.21 ^NS^	14.96 ± 1.21 ^NS^	-	1
	IF	22.38 ± 3.49	4.54 ± 1.44	14.30 ± 0.64	-	1
	8 DAF	20.95 ± 5.85	5.73 ± 1.13	12.94 ± 1.17	1.50 ± 0.75 ab	1
	16 DAF	21.88 ± 3.10	5.29 ± 2.61	13.56 ± 1.24	4.93 ± 1.85 a	2
	24 DAF	25.70 ± 5.01	3.43 ± 0.63	14.55 ± 0.52	4.00 ± 1.04 a	2
	32 DAF	20.93 ± 3.96	7.26 ± 2.04	14.00 ± 0.77	0.54 ± 0.33 b	1
	40 DAF	19.19 ± 6.24	6.52 ± 3.32	13.63 ± 1.68	0.97 ± 0.73 b	1
C.V.		22.98	35.82	9.19	46.61	
IPR Sabiá	Control	13.97 ± 3.37 ab	9.76 ± 2.39 ^NS^	18.41 ± 1.09 ^NS^	-	1
	IF	17.71 ± 2.44 a	7.95 ± 1.76	17.04 ± 2.26	-	1
	8 DAF	16.69 ± 2.43 ab	6.91 ± 1.39	16.86 ± 1.07	0.85 ± 0.62 ^NS^	1
	16 DAF	9.72 ± 1.40 b	10.86 ± 1.95	18.40 ± 1.60	1.76 ± 0.77	1
	24 DAF	9.76 ± 2.50 b	8.63 ± 0.90	17.04 ± 1.64	2.66 ± 0.86	2
	32 DAF	13.86 ± 3.22 ab	9.22 ± 1.23	18.74 ± 1.02	0.88 ± 0.48	1
	40 DAF	20.20 ± 2.50 a	10.32 ± 1.93	18.31 ± 0.91	1.12 ± 0.69	1
C.V.		17.33	18.47	16.11	41.02	
IPR Urutau	Control	13.01 ± 2.99 ^NS^	11.37 ± 2.63 ^NS^	13.36 ± 1.58 ^NS^	-	1
	IF	13.89 ± 5.35	7.43 ± 2.02	12.72 ± 0.80	-	1
	8 DAF	18.27 ± 5.31	5.92 ± 2.83	14.20 ± 0.79	2.43 ± 1.21 ab	1
	16 DAF	18.16 ± 2.72	5.03 ± 1.34	12.01 ± 1.59	3.95 ± 0.66 a	2
	24 DAF	10.06 ± 4.75	9.59 ± 1.63	15.70 ± 1.21	0.88 ± 0.68 ab	1
	32 DAF	12.63 ± 3.92	7.63 ± 1.68	13.42 ± 2.03	0.42 ± 0.42 ab	1
	40 DAF	17.06 ± 3.36	8.64 ± 0.91	13.10 ± 1.53	0.00 ± 0.00 b	1
C.V.		33.74	27.57	12.49	40.88	

Note: means ± standard error followed by the same letter in the column (for each cultivar) do not differ statistically following Tukey’s test (α = 0.05); ^NS^ = not significant; IF = initial flowering; DAF = days after flowering; Type = classification based on the Instrução Normativa # 12/2008 do Ministério da Agricultura e Pecuária.

## Data Availability

The raw data supporting the conclusions of this article will be made available by the authors on request.
